# In Search of Risk Factors for Recurrent Erysipelas and Cellulitis of the Lower Limb: A Cross-Sectional Study of Epidemiological Characteristics of Patients Hospitalized due to Skin and Soft-Tissue Infections

**DOI:** 10.1155/2020/1307232

**Published:** 2020-05-07

**Authors:** Mariusz Sapuła, Dagny Krankowska, Alicja Wiercińska-Drapało

**Affiliations:** Department of Infectious and Tropical Diseases and Hepatology, Medical University of Warsaw, Hospital for Infectious Diseases, Warsaw, Poland

## Abstract

**Background:**

Erysipelas and cellulitis are common, acute, bacterial infections of the skin and subcutaneous tissue. The incidence of these infections is growing, and the recurrence rate is high. Effective antibiotic prophylaxis is available, but insufficient data exist on the risks factors for recurrent infection.

**Purpose:**

To compare comorbidities and laboratory findings in patients with single-episode and recurrent erysipelas/cellulitis in order to identify risk factors for recurrent erysipelas/cellulitis.

**Methods:**

A cross-sectional study, which included patients hospitalized in the Department of Infectious and Tropical Diseases and Hepatology of the Medical University of Warsaw due to erysipelas and cellulitis during 3 consecutive years (July 2016–June 2019).

**Results:**

The study included 163 patients, of which 98 had a first episode of erysipelas/cellulitis and 65 had a recurrence. The recurrent infection was significantly associated with a history of lymphedema (12.3% in the recurrent group vs. 2.0% in the first-episode group, *p*=0.015), a higher BMI (35.4 vs. 31.2, respectively, *p*=0.002), chronic obstructive pulmonary disease (10.8% vs. 2.0%, *p*=0.030), and a shorter history of symptoms prior to hospitalization (6.0 days vs. 11.8 days, *p*=0.004). Patients with the first episode of infection were more likely to have had minor local trauma directly preceding the symptoms of infection (20.4% in the first-episode group vs. 1.5% in the recurrent group, *p*=0.001).

**Conclusions:**

Patients with lymphedema and obesity should be viewed at high risk of developing recurrence of erysipelas and thus should be considered as candidates for antibiotic prophylaxis and other prevention methods. Minor local trauma directly preceding the skin infection does not by itself confer a higher risk for erysipelas recurrence. More research is needed to assess the association of recurrent skin and soft-tissue infection to preceding minor local trauma, individual components of the metabolic syndrome, and COPD.

## 1. Introduction

Erysipelas and cellulitis are common, acute, bacterial infections of the skin and underlying soft tissue. Traditionally, erysipelas was distinguished from cellulitis by a more distinct, elevated border and it was presumed to be different in etiology, but recently this distinction has been put into question by many authors [1–3].

It is frequently assumed that erysipelas and cellulitis are caused by group A streptococci and *Staphylococcus aureus*, but other streptococci and Gram-negative bacteria seem to be the causative agents in some cases [2–4]. These infections usually affect the lower limb and can be diagnosed clinically in patients presenting with sudden onset of pain, edema, erythema, and increased warmth of affected localization, as well as systemic symptoms such as fever and chills. Bullae may also appear on the skin. Laboratory findings are nonspecific and may involve increased white blood cell count with a predominance of neutrophils and an elevated C-reactive protein.

Some studies show an increase in the incidence of erysipelas and cellulitis, and in European countries, it is estimated to be 200 per 100,000 people per year [5].

Various risk factors for the initial episode of erysipelas and cellulitis of the lower limb were found in previous studies: local edema/lymphedema, venous insufficiency, disruption of the cutaneous barrier, and being overweight [6, 7].

Patients with these infections can usually be successfully treated with narrow-spectrum penicillins, but sometimes require hospitalization and intravenous treatment. The most common complication of erysipelas is recurrence of the infection. The recurrence occurs in up to 41% of patients in 5 years [8].

A number of interventional studies focused on pharmacological prophylaxis of recurrence. Most of these included patients with at least two episodes of infection in the span of 3 years and evaluated narrow-spectrum antibiotics, mainly penicillin, which seemed to be effective for the duration of the prophylaxis [9]. Some demonstrable causes of penicillin prophylaxis failure include noncompliance and erysipelas due to resistant pathogens (such as MRSA or Gram-negative bacteria); incorrect dose and selecting an antibiotic without proven efficacy in this setting are other possible causes enumerated by Koster et al. [10].

Although effective antibiotic prophylaxis for recurrent prophylaxis is available, insufficient data exist on the risks factors for recurrent infection, and so it may be difficult to predict whether a given patient is a good candidate for such interventions.

## 2. Aim

To compare demographic characteristics, comorbidities, and laboratory findings in patients with first-episode erysipelas and recurrent erysipelas in order to identify risk factors for recurrent erysipelas.

## 3. Materials and Methods

Medical records of patients hospitalized in the Department of Infectious and Tropical Diseases and Hepatology of the Medical University of Warsaw, Poland, due to erysipelas or cellulitis during 3 consecutive years (July 2016–June 2019) were analyzed in the study. For the purposes of this study, no distinction was made between patients with erysipelas and cellulitis (henceforth referred to collectively simply as “erysipelas”) as there was no difference in the management of this condition in our clinical center. Patients with erythematous and bullous erysipelas were included in the study, but those with necrotizing fasciitis and osteomyelitis were excluded. Only patients with erysipelas of the lower limb were included.

Demographic, clinical, and laboratory markers were recorded into a database, and the data were analyzed using statistical software to compare patients with first-episode and recurrent erysipelas.

If more than one record of a recurrent episode of a particular patient was present in the database, only the first one was included into the analysis.

For data analysis, the chi-squared test (or Fisher's exact test, if applicable) and Student's *t*-test were used in a univariate analysis to compare the qualitative and quantitative variables between groups. The *R* statistical analysis software was used for all calculations.

## 4. Results

### 4.1. Sample Baseline Characteristics

A total of 163 patients hospitalized with erysipelas of the lower extremity were identified during 3 consecutive years (July 2016–June 2019). The mean age was 66.1 years (median age was 65 years), and 47.2% were female. There were 98 patients with single-episode erysipelas (SE) and 65 patients with recurrent erysipelas (RE). The median number of episodes for the recurrent erysipelas group was 3, ranged from 2 to more than 10.  Table 1 shows the distribution of the number of erysipelas episodes.

Blood cultures on admission were performed in 40 patients (24.5% of total) and were positive in 2 patients (5.0% of tested patients)—one culture yielded *Streptococcus pyogenes* and the other one *Streptococcus sp.* pertaining to group C.

5 patients from the recurrent group (7.7%) were on antibiotic erysipelas prophylaxis at the time of recurrence.

Only one patient was found to have concomitant deep venous thrombosis of the affected limb and one patient developed a minor skin abscess, although ultrasound of the affected limb was performed only when clinically indicated (22.3% of cases).

### 4.2. Comparison between Single-Episode Erysipelas and Recurrent Erysipelas

There was no difference between the age (*p*=0.076) nor gender distribution (*p*=0.386) between the two groups (Table 2). Patients with RE had a significantly higher body mass index (BMI) (35.4 vs. 31.2; *p*=0.002), and they were more likely to have a history of lymphedema (12.3% vs. 2.0%; *p*=0.015) as well as residual lymphedema at discharge (15.4% vs. 5.1%; *p*=0.026) than patients with SE (Tables 3 and 4). The only chronic illness that showed a significant association with RE was chronic obstructive pulmonary disease (COPD) (10.8% vs 2.0%; *p*=0.030) (Table 3).

Trauma preceding the development of erysipelas was much more common in SE than in RE (10.8% vs. 2.0%; *p*=0.030) (Table 4).

Laboratory findings were compared between patients with SE and RE, but no significant differences were observed (Table 5). The length of stay in the hospital was similar for both groups. Patients who had the first episode of erysipelas had a longer history of symptoms preceding hospital admission in comparison with those who had a recurrent episode (11.8 days vs. 6.0 days; *p*=0.004) (Table 6).

As lymphedema can lead to confounding when comparing the BMI of both groups, a separate comparison was done with a subgroup of patients without a history of lymphedema (Table 7). No inferential statistics were done on the subgroup with a history of lymphedema, as the number of cases in this subgroup was too small (*n* = 10).

## 5. Discussion

In our study, similarly as in Inghammar et al. [11] erysipelas recurrence was significantly associated with increased BMI, but not with other comorbidities associated with the metabolic syndrome (hypertension, diabetes mellitus, or dyslipidemia). In a study by Brishkoska-Boshkovski et al. [3], in addition to an association between obesity (but no differences in mean BMI) and RE, the recurrence was also linked to diabetes mellitus treated with insulin. Obesity and diabetes were also independent predictors of lower limb cellulitis recurrence in a large longitudinal cohort study by Cannon et al. [12]. In a study by Kozłowska et al., being overweight and having hypertension but not diabetes were more prevalent in patients with recurrent erysipelas [13]. On the other hand, Karppelin et al. did not find any association between components of the metabolic syndrome and erysipelas recurrence [8]. The association between the metabolic syndrome and erysipelas needs further research.

Apart from obesity, the only chronic disease that was positively associated with recurrent erysipelas in our study was chronic obstructive pulmonary disease (COPD). This is in contrast to the results of Inghammar et al. where COPD was more prevalent in first-episode erysipelas. Inghammar et al. argued that this might be due to the high use of antibiotics against respiratory infections in this group of patients and that it might cause a decrease of colonizing agents responsible for erysipelas. Cannon et al. also mention chronic pulmonary disease as an independent risk factor for RE, but the authors do not specify which chronic diseases they mean [12].

Some authors stress the importance of toe web dermatophytosis as a risk factor for erysipelas [6, 8, 14]. Due to the retrospective character of our study, certain medical records lacked the information about the presence or absence of toe web intertrigo; thus, we did not include this potential risk factor in the statistical analysis of this study.

Both in this and previous studies, minor trauma was seen mainly in the setting of first-episode erysipelas [3, 8, 11, 14]. One possible interpretation is that the disruption of the skin barrier predisposes to a single, nonrecurrent episode of erysipelas, whereas the recurrence of infection is facilitated mainly by chronic risk factors. On the other hand, one episode of erysipelas may itself be a risk factor for a repeated episode of infection, and thus some patients with a recent history of minor trauma could still be at risk for recurrence. Furthermore, research is required to support this hypothesis.

Lymphedema is an established risk factor for RE and frequent complication of erysipelas [3, 8, 11, 12]. Our data are consistent with previous studies, as patients with RE were more likely to have a history of lymphedema (12.3% vs. 2.0%; *p*=0.015) than patients with SE. As Chlebicki et al. pointed out in their paper, a vicious circle is created with lymphedema being both the complication of erysipelas and an important risk factor for recurrence [15].

We found no differences in the studied laboratory parameters between groups, as opposed to Brishkoska-Boshkovski et al. [16].

There are some limitations in this study. This study was not prospective in nature, and thus there is a possibility of bias: the patients included in the first-episode group might in the future have a recurrence, thus biasing the result in the direction of possibly missing a statistically significant association. Another possible limitation is the fact that the studied population was comprised of hospitalized patients, and thus the conclusions of this study may not be applicable to patients with erysipelas treated in the outpatient clinic. In spite of the above, we believe that the study design and its conclusions are appropriate and valid given the clinical problem that we aimed to explore in this study.

## 6. Conclusions

Patients with lymphedema and obesity should be viewed at high risk of developing recurrence of erysipelas and thus should be considered as candidates for antibiotic prophylaxis and other prevention methods. Minor local trauma directly preceding the skin infection does not by itself confer a higher risk for erysipelas recurrence. More research is needed to assess the association of preceding minor local trauma, individual components of the metabolic syndrome, and COPD to recurrent skin and soft-tissue infection.

## Figures and Tables

**Table 1:** The distribution of the number of erysipelas episodes for the recurrent group.

Number of episodes	2	3	4	5	6	7	8	9	≥10
Number of patients (percentage of patients)	23 (35%)	10 (15%)	10 (15%)	12 (19%)	2 (3%)	2 (3%)	1 (2%)	0 (0%)	5 (8%)

**Table 2:** Association of demographic characteristics and recurrent lower extremity erysipelas.

Parameter	First episode	Recurrent episode	*p*
Gender, female	50.0%	43.6%	0.386
Age (years)	67.9 +/− 16.7	63.4 +/− 14.8	0.076
Homelessness	4.1%	4.6%	1.000

Age shown as mean +/− standard deviation.

**Table 3:** Association of comorbidities and recurrent lower extremity erysipelas.

Comorbidity	First episode	Recurrent episode	*p*
BMI (kg/m^2^)	31.2 +/− 7.7	35.4 +/− 8.9	0.002
Hypertension	69.4%	69.2%	0.983
Diabetes mellitus	26.5%	33.8%	0.316
Dyslipidemia	28.6%	24.6%	0.577
Congestive heart failure	32.7%	33.8%	0.874
Chronic kidney disease	12.2%	9.2%	0.548
Chronic obstructive pulmonary disease	2.0%	10.8%	0.030
Immunodeficiency	9.1%	1.5%	0.052
Cancer, history	8.2%	4.6%	0.528
Cancer, present	0.5%	0.2%	0.404
History of stroke	5.1%	4.6%	1.000
History of myocardial infarction	7.1%	3.1%	0.319

Quantitative variables are shown as mean +/− standard deviation.

**Table 4:** Association of local risk factors and recurrent lower extremity erysipelas.

Local risk factor	First episode	Recurrent episode	*p*
Chronic ulcer	9.2%	15.4%	0.227
Chronic dermatological disease	12.2%	7.7%	0.352
Minor local trauma	20.4%	1.5%	0.001
History of lymphedema	2.0%	12.3%	0.015
Lymphedema at discharge	5.1%	15.4%	0.026

**Table 5:** Association of laboratory parameters and recurrent lower extremity erysipelas.

Parameter	First episode	Recurrent episode	*p*
Hemoglobin (g/dL)	12.7 +/− 1.9	13.1 +/− 1.4	0.092
Platelet count (G/L)	228 +/− 93	243 +/− 104	0.358
White blood cell count (G/L)	10.9 +/− 4.9	10.8 +/− 4.3	0.885
Neutrophil count (G/L)	8.3 +/− 4.7	8.4 +/− 4.4	0.854
C-reactive protein (mg/l)	130 +/− 111	114 +/− 97	0.335
Procalcitonin (ng/ml)	2.97 +/− 7.62	1.20 +/− 1.83	0.152
D-dimers (ng/ml)	1618 +/− 1243	1387 +/− 1058	0.273
Urea (mmol/l)	8.3 +/− 5.5	7.3 +/− 2.9	0.199
Creatinine at admission (umol/l)	97 +/− 67	92 +/− 47.1	0.598
Creatinine at discharge (umol/l)	73.9 +/− 21.5	72.9 +/− 19.5	0.764

Values are shown as mean +/− standard deviation.

**Table 6:** Association of other parameters and recurrent lower extremity erysipelas.

Parameter	First episode	Recurrent episode	*p*
Oral antibiotics prior to admission	39.8%	26.2%	0.073
Hospitalisation duration (days)	15.8 +/− 7.1	14.5 +/− 6.1	0.205
Symptoms' duration prior to admission (days)	11.8 +/− 17.5	6.0 +/− 7.9	0.004
Bilaterality of erysipelas	9.2%	7.7%	0.739
Bullous presentation	25.5%	15.4%	0.123

Quantitative variables are shown as mean +/− standard deviation.

**Table 7:** Association of BMI and recurrent lower extremity erysipelas in patients without lymphedema: 96 patients with first episode and 57 patients with recurrence.

Parameter	First episode	Recurrent episode	*p*
BMI (kg/m^2^)	31.4 +/− 7.2	34.0 +/− 8.9	0.035

BMI value is shown as mean +/− standard deviation.

## Data Availability

The data generated and/or analyzed during the current study are available from the author at mariusz.sapula@gmail.com upon reasonable request.
